# How Sex of Children with Autism Spectrum Disorders and Access to Treatment Services Relates to Parental Stress

**DOI:** 10.1155/2014/721418

**Published:** 2014-12-14

**Authors:** Irina Zamora, Eliza K. Harley, Shulamite A. Green, Kathryn Smith, Michele D. Kipke

**Affiliations:** ^1^Keck School of Medicine, University of Southern California, Los Angeles, CA 90089, USA; ^2^USC University Center for Excellence in Developmental Disabilities, Children's Hospital Los Angeles, 4650 Sunset Boulevard, MS No. 53, Los Angeles, CA 90027, USA; ^3^Rady Children's Hospital San Diego, 3020 Children's Way, MC 5023, San Diego, CA 92123, USA; ^4^University of California, Los Angeles, Semel Institute for Neuroscience and Human Behavior, Ahmanson Lovelace Brain Mapping Center, 660 Charles E. Young Drive South, Los Angeles, CA 90095, USA

## Abstract

Parents of children with autism spectrum disorders (ASD) experience higher levels of stress in comparison to parents of neurotypical children and consequently are more susceptible to negative health and social outcomes (Dunn et al., 2001). However, less is known about how individual child characteristics impact stress levels in parents of children with ASD. In this study, we examined the relationship between individual characteristics (i.e., sex) of children with ASD and parental stress. Access to comprehensive treatment services was also examined as a contributing factor to parental stress. Parenting stress was higher for parents of girls than for parents of boys, and for parents of girls (but not boys) fewer services predicted higher parental distress. Findings highlight the importance of providing parents of girls with ASD with more tailored support.

## 1. Introduction

Parents of children with autism spectrum disorders (ASD) experience higher levels of stress in comparison to parents of neurotypical children and consequently are more susceptible to negative health and social outcomes [1]. Specifically, parents of children with ASD are more likely to experience depression, anxiety, somatic complaints, isolation, and burnout [2]. However, most research related to parental stress and developmental disability has focused on mothers. Tehee et al. [3] found that mothers of children with ASD experience higher level of stress than fathers, and stress in mothers was found to be most connected to the following four areas: parenting problems, level of child self-sufficiency, behavior, and physical development [4].

Previous research also suggests that parental stress is often correlated with child characteristics, locus of parenting control, parenting satisfaction, and social support [5]. Additionally, challenges in obtaining a timely ASD diagnosis and lack of appropriate treatment services and education are factors identified in the literature as contributors to parental stress and dissatisfaction [6, 7]. In fact, mean time to diagnosis in children is approximately 2-3 years [8]. However, less is known regarding how individual child characteristics, access to comprehensive treatment services, and individual functioning intersect to impact stress levels in parents of children with ASD.

The sex ratio in ASD is one of the most consistent findings in the field, with the condition more commonly identified in boys, 5 times more common among boys (1 in 49) than girls (1 in 189 [9]). The evidence for sex differences in presentation between boys and girls with ASD is mixed. However, previous research has acknowledged that girls may present differently than boys, making timely diagnosis more difficult given our current understanding and availability of diagnostic instruments [10].

Research has shown that early intervention is associated with better outcomes for children with ASD [11]. However, if a child is identified later in life, early intervention is less likely to occur, possibly impacting the outcome of treatment services. Consequently, parents of female children with ASD may experience challenges not only in obtaining a timely and accurate diagnosis, but also in connecting to treatment services and identifying sources of social support.

Social support, both within and outside of the family, has been correlated with parental stress [5]. More specifically, low levels of within-family support have been associated with higher levels of psychological distress for families who have children with ASD. On the other hand, outside support is consistently tied to increased parental well-being, and this type of informal social support appears to be especially significant for mothers of children with ASD [12].

Given the aforementioned considerations, we examined how parental stress varies by child characteristics (i.e., sex) and intersects with access to comprehensive treatment services. We expected to find that families of female children with ASD have less access to services, and we predicted that fewer services would be associated with greater parental stress.

## 2. Methods

### 2.1. Participants and Procedures

All study participants were recruited between 2010 and 2013 through the Autism Speaks Autism Treatment Network (AS/ATN) at Children's Hospital Los Angeles. The study included 166 participants, parents of 27 girls and 89 boys between the ages of 1 to 15 years. Over half of the participants were from an ethnic minority background. All of the parents who participated in the study were mothers. Demographic information is presented in Table 1. All of the children in the study met diagnostic criteria for autistic disorder, Asperger's disorder, or pervasive developmental disorder-not otherwise specified (PDD-NOS), according to the DSM-IV-TR [13]. Additionally, the participants' scores on the Autism Diagnostic Observation Schedule [14] had to meet or exceed cutoffs for categorization of autism spectrum disorder. Information from the* Parenting Stress Index 3, Short Form* (PSI-3-Short Form [15]), and the medical form was extracted to obtain information about self-reported parental stress and services that children were receiving at the time of the ATN enrollment.

### 2.2. Measures

#### 2.2.1. Parental Functioning

The* Parenting Stress Index 3, Short Form* (PSI-3-Short Form [15]), is a 36-item subset of the 120-item full-length measure. This parent-report questionnaire is designed for parents of children aged birth to 12 years. It assesses the parent's level of stress in the parent-child relationship, as well as the level of parental distress (parental distress (PD)), dysfunctional interaction (parent-child dysfunctional interaction (P-CDI)), and parenting difficulty associated with the child (difficult child (DC)). This measure has been shown to have good reliability and validity and has been used in a variety of child health and parenting studies to assess parental stress and to assess levels of stress in families with a child with a developmental disability [5, 16].

#### 2.2.2. Child Functioning

*Autism Diagnostic Observation Schedule*. The Autism Diagnosis Observation Schedule, Generic (ADOS-G; [14]) is a semistructured, standardized assessment of communication, reciprocal social interaction, and imaginative play. The ADOS is currently considered a gold standard for assessing autism. It consists of standardized activities that allow the examiner to observe behaviors that have been identified as important to the diagnosis of autism spectrum disorders. The ADOS is composed of 4 modules, which are completed based on the child's language skills. All of the participants met criteria for an ASD diagnosis based on ADOS outcomes and the clinical judgment of a licensed clinical psychologist with expertise in the area of ASD and DSM-IV-TR criteria. The ADOS social-communication score was standardized to allow for combined analysis of participants completing different modules.

#### 2.2.3. Child Services

Parents completed a Medical History form, which included information about the autism-related treatment services (e.g., school services, therapies, and Regional Center services) that children were receiving at the time of AS/ATN enrollment. The treatment services were grouped as follows: (1) school services, which included autism classroom, special education school, and learning center/resource room services; (2) Regional Center services—nonprofit private organization in California that provides services and support to individuals with developmental disabilities and their families—which included behavioral therapy, Floortime, and other social skills training; (3) mental health therapy, which included individual psychotherapy and family therapy; and (4) speech and language therapy services. Children received a score of 1 or 0 in each service category depending on whether or not they received services of that kind. The service types were summed to create a total services variable; a score of 0 for this variable indicated that a child was not receiving services in any of the categories, and a maximum score of 4 indicated that the child was receiving services in all of the categories.

## 3. Results

### 3.1. Descriptive Statistics

Demographic variables and main study variables are displayed in Table 1. Statistics are displayed both for the total sample and for males and females separately. In the total sample, almost half the children were receiving speech and mental health services, whereas a minority were receiving school or Regional Center services.

### 3.2. Sex Differences

Independent-sample *t*-tests and Chi-square analyses were used to test for gender differences in demographic and main study variables. Females were significantly older and less likely to be in a minority group than males. There were no significant gender differences in percent of any of the services received, although girls qualitatively appeared to be receiving fewer speech and Regional Center services than boys but more school services; the lack of significance may be due to the relatively small size of the female group. Parents of girls rated parental distress and parent-child dysfunctional interaction significantly higher than parents of boys. There were no significant group differences in severity of autism symptoms.

### 3.3. Total Services as a Predictor of Parental Stress

Hierarchical regressions were performed to examine whether total number of services predicted parental stress over and above child demographic variables. Four regression models were examined to predict each of the parenting stress subscales (difficult child, parent-child dysfunctional interaction, and parental distress) and the total stress score. Child gender, child age, family income, child minority status, and ADOS social-communication scores were entered in the first step. Covariates that were significant at *P* > 0.10 were removed from the final model to conserve power. Step 2 included total number of services received, and Step 3 included a gender by services interaction term to determine whether services predicted parental stress differently for girls compared to boys. Results of the four regression models are displayed in Table 2.

Child gender predicted all three subscales (but not the total stress scale) with parents of girls reporting significantly more stress than parents of boys. Age predicted only P-CDI; parents of older children reported higher P-CDI stress. ADOS severity predicted both difficult child and parental distress scores, although, surprisingly, the direction of effect was different. Higher ADOS severity predicted lower difficult child scores but higher parental distress scores.

There were no significant main effects for total services. However, there was a significant gender by services interaction predicting parental distress, such that fewer services predicted higher stress for parents of girls but not for parents of boys.

### 3.4. Relationship between Individual Services and Parental Distress

Because services significantly predicted parental distress scores, we followed up on the regression analyses by examining the relationship between parental distress and each individual service type using univariate analysis of variance (ANOVA). Four ANOVAs were conducted, each examining the main effect of gender and service type on parental distress as well as the gender by service type interaction (see Table 3). ADOS severity scores and age were included as covariates; age was removed from the final models as it was not significant in any model.

**Figure 1:**
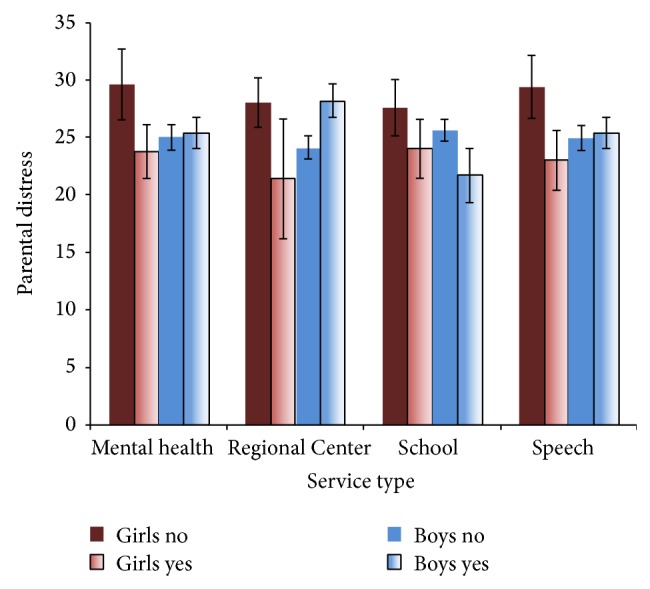
Parental distress scores for boys and girls by service type. Note: error bars indicate standard error of the mean. *N* = 27 girls, 89 boys.

There was no main effect for gender or service type in any model. There was a significant gender by service interaction for Regional Center, with Regional Center services predicting lower parental distress for girls but higher for boys. There was also a marginally significant gender by service interaction for speech and mental health, with parents of girls with speech or mental health services having lower parental distress than those without these services (at the trend level only). Parental distress scores by service type are displayed in Figure 1.

## 4. Discussion

This study examined the relationship between parental stress and access to services in families of children with ASD, as well as how this relationship differed by the sex of the child. Parents of girls in our sample rated parental distress and parent-child dysfunctional interaction significantly higher than parents of boys. We did not find a main effect of access to services on parental stress; however, access to services did predict higher parental stress specifically for parents of girls.

The fact that girls who received Regional Center services predicted lower parental distress for parents of girls with ASD demonstrates the importance of obtaining early comprehensive treatment services for females with ASD, especially because Regional Center services are often most easily obtained before the age of 3 years. Additionally, decreasing parental stress may have an effect on the impact of intervention on the child, especially for girls. Mothers who report higher levels of self-efficacy have been found to assume a more active role in promoting their child's development and demonstrate increased collaboration with the child's intervention team [17, 18].

While not being the focus of the study, we also found that autism symptom severity was predictive of both parental stress and access to services. Not surprisingly, parents of children with more severe symptoms reported higher levels of parental stress overall, and their children had more total services. Interestingly, parents of children with more severe symptoms of ASD reported their children as less difficult but reported higher levels of parental distress. It may be that these parents do not characterize their children as “difficult” as they attribute their behavior to their autism severity, but they still experience high levels of distress around their child's behavior.

The implications of this study provide insight for providers working with children with ASD and their parents. Understanding and targeting parental stress is critical in enhancing well-being and the parent-child relationship. This is especially important for parents of girls with ASD because they may lack services within mental health such as family or dyadic therapy that targets and addresses parental stress. Furthermore, difficulty in identifying girls with ASD and/or targeting their specific needs may generate additional parental stress.

This study also contains limitations. First, the relatively small sample size of the female group may account for reduced power and a lack of significant gender differences in overall intervention services received. For this study, we lacked data in regard to cognitive and behavioral functioning. Additionally, the data were collected at the time of intake and changes in services after assessment were not analyzed in this study.

Future research comparing services before and after assessment is recommended to better understand the impact of our services and support in regard to gender differences, parental stress, and access to services. Future research should continue to focus on specific challenges experienced by parents of female children with ASD, including ways to increase access to comprehensive treatment services and the prevalence of educational and social support. Differences in parental stress experienced between mothers and fathers should also be examined.

## Figures and Tables

**Table 1:** Descriptive statistics.

	Total sample percentage or mean (SD)	Boys percentage or mean (SD)	Girls percentage or mean (SD)	*χ* ^2^ (df = 1) or *t* (df = 116)	*F* (Levine's test for equality of variances)
Child gender (% male)	78%	—	—	—	—
Child age (in years)	5.36 (3.34)	4.97 (3.10)	6.67 (3.83)	2.33^*^	3.90^+^
Percent Hispanic^a^	30.3%	27.7%	38.5%	1.08	—
Child race (% minority)	58.3%	64.0%	38.5%	11.62^*^	—
Family income (% <$ 50 k)^b^	23.0%	24.6%	21.4%	3.64	—
Speech services (% yes)	48.7%	51.7%	38.5%	1.41	—
School services (% yes)	13.9%	11.2%	23.1%	2.36	—
Regional Center services (% yes)	23.5%	25.8%	15.4%	1.23	—
Mental health services (% yes)	45.2%	44.9%	46.2%	0.01	—
Total services	1.30 (1.06)	1.33 (1.05)	1.23 (1.11)	−0.40	0.62
PSI difficult child	32.95 (9.89)	31.74 (8.83)	37.08 (12.19)	2.08^*^	7.37^**^
PSI parent-child dysfunctional relationship	25.17 (7.69)	24.07 (7.17)	28.92 (8.35)	2.93^**^	3.47^+^
PSI parent distress	25.66 (8.54)	25.17 (8.02)	27.35 (10.09)	1.15	1.82
PSI total score	87.82 (23.14)	86.60 (21.54)	92.00 (28.03)	1.10	3.90^+^
ADOS social-communication composite	52.21 (22.73)	53.76 (21.78)	46.88 (25.47)	−1.36	0.94

^+^ *P* < 0.10.^*^ *P* < 0.05. ^**^ *P* < 0.01.

Note: *N* = 27 girls, 89 boys except where otherwise noted.

^a^ *N* = 27 girls, 83 boys; 6 participants did not report ethnicity.

^b^ *N* = 15 girls, 57 boys; 44 participants did not report income.

**Table 2:** Regression analysis predicting parenting stress from total number of services received.

PSI model	Difficult child		P-CDI		Parental distress		Total stress	
	*β*	Δ*R* ^2^	*β*	Δ*R* ^2^	*β*	Δ*R* ^2^	*β*	Δ*R* ^2^
Step 1: child covariates	—	0.09^**^	—	0.11^**^	—	0.04^+^	—	—
Gender	−0.33^*^	—	−0.31^*^	—	−0.38^**^	—	—	—
Age	—	—	0.20^*^	—	—	—	—	—
ADOS social-communication	−0.19^*^	—	—	—	0.19^*^	—	—	—
Step 2: total services	−0.30	0.01	−0.21	0.01	−0.44^*^	0.00	0.09	0.00
Step 3: gender × services interaction	0.28	0.01	0.18	0.01	0.51^*^	0.05^*^	−0.14	0.01

^+^ *P* < 0.10. ^*^ *P* < 0.05. ^**^ *P* < 0.01.

**Table 3:** ANOVA examining differences in parental distress based on receipt of each service type.

Service type	Mental health		Regional Center		School		Speech	
	MS	*F*	MS	*F*	MS	*F*	MS	*F*
ADOS social-communication (df = 1)	379.32	5.38^*^	275.10	3.96^*^	297.20	4.15^*^	358.48	5.09^*^
Gender (df = 1)	94.40	1.34	5.24	0.08	73.40	1.03	54.42	0.77
Service type (df = 1)	189.36	2.68	17.13	0.25	144.97	2.03	222.53	3.26^+^
Gender × service type (df = 1)	224.01	3.18^+^	319.54	4.60^*^	0.57	0.01	225.12	3.20^+^
Error (df = 111)	70.55	—	69.43	—	71.59	—	0.09	0.00

^+^ *P* < 0.10. ^*^ *P* < 0.05.
